# The effects of art therapy interventions on anxiety in children and adolescents: A meta-analysis

**DOI:** 10.1016/j.clinsp.2024.100404

**Published:** 2024-06-26

**Authors:** Bo Zhang, Jiahua Wang, Azizah binti Abdullah

**Affiliations:** a Guizhou Equipment Manufacturing Polytechnic, Guizhou, China; b Universiti Utara Malaysia, Malaysia

**Keywords:** Anxiety, Children, Adolescents, Art therapy, Meta-analysis

## Abstract

**Objective:**

This study aimed to assess the effects of art therapy on anxiety among children and adolescents.

**Methods:**

We searched several databases, including PubMed, Web of Science, Embase (via Ovid), PsychINFO (through EBSCO), and The Cochrane Library, comprising the Cochrane Database of Systematic Reviews and the Cochrane Central Register of Controlled Trials. Additionally, Chinese databases such as CNKI (China National Knowledge Infrastructure) and Wan Fang Data were explored from their beginnings until October 22, 2023. Studies that investigated the impact of art therapy on anxiety compared to a control group were included. The methodological quality of these randomized controlled trials was evaluated using the Cochrane Handbook's risk of bias instrument.

**Results:**

Six studies involving 422 participants were included. The findings indicated a notable decrease in anxiety symptoms due to art therapy, with a Standardized Mean Difference (SMD) of -1.42, 95% Confidence Interval (95%CI -2.33, -0.51), p < 0.002. Notably, there was pronounced heterogeneity, as evidenced by Tau^2^ = 1.41, Chi^2^ = 101.19, df = 6, and I² = 94%, with Z = 3.06.

**Conclusion:**

Art therapy significantly improved the anxiety symptoms of children and adolescents, positioning it as an effective means of treating anxiety.

## Highlights

• This meta-analysis aimed to evaluate the impact of art therapy on reducing anxiety symptoms among children and adolescents. A comprehensive search was conducted across several databases, including PubMed, Web of Science, Embase, PsychINFO, The Cochrane Library, CNKI, and Wan Fang Data, resulting in the inclusion of six studies with a total of 422 participants. The findings demonstrated a significant decrease in anxiety symptoms through art therapy interventions, with a Standardized Mean Difference (SMD) of -1.42, 95% Confidence Interval (-2.33, -0.51), p < 0.002. This indicates that art therapy is an effective method for treating anxiety in this demographic. Moreover, the analysis revealed that art therapy had a more pronounced effect on state anxiety than on trait anxiety, suggesting that art therapy may be particularly beneficial in helping children and adolescents manage anxiety in specific situations. These insights underscore the importance of integrating art therapy into mental health care, especially for managing anxiety among young individuals, and have significant implications for clinical practice and policy making.

## Introduction

Anxiety stands as one of the predominant mental health issues influencing an individual's emotions, cognition, behavior, and physiological responses.^1^ Given that children and adolescents undergo swift developmental transitions, encountering a myriad of stresses and adversities, they represent a particularly vulnerable demographic to anxiety's ramifications.^2^, ^3^, ^4^ Global estimates suggest that 6.5% of children and adolescents worldwide suffer from anxiety disorders.^5^ Notably, the surge of the 2019 coronavirus pandemic has exacerbated anxiety levels among these young individuals.^6^^,^^7^ Beyond diminishing quality of life and academic achievements, anxiety amplifies the potential for other mental health complications and elevates suicide risks.^8^, ^9^, ^10^ The pervasive detrimental effects of anxiety during youth, combined with insights that most anxiety disorders first emerge during these formative years, emphasize the imperative of tailored interventions for this age group, encompassing both therapeutic and preventative measures.^11^, ^12^, ^13^

For anxiety disorders, prevailing treatment modalities encompass Cognitive Behavioral Therapy (CBT) and a range of pharmacotherapies.^14^, ^15^, ^16^, ^17^ Nonetheless, these endorsed strategies frequently exhibit limited effectiveness. It is striking that between 20%‒50% of patients diagnosed with anxiety disorders either present contraindications or do not experience marked improvement with these treatment approaches.^18^, ^19^, ^20^, ^21^ While some research advocates for a combined regimen of pharmacotherapy and CBT,^22^ the reality remains that nearly half of the patient cohort exhibits a suboptimal response to CBT alone.^23^

In the quest for effective treatment strategies for anxiety disorders, clinicians have embraced innovative methodologies to augment therapeutic outcomes. Prominently, art therapy has garnered increasing interest, and it has been assimilated into various mental health regimens for individuals grappling with anxiety.^24^^,^^25^ Furthermore, it can be employed as an independent therapeutic intervention.^26^, ^27^, ^28^ While art therapy is perceived as a valuable supplementary treatment for diverse mental ailments,^27^, ^28^, ^29^, ^30^ a definitive body of scientific evidence supporting its efficacy remains elusive.

In art therapy, practitioners employ visual mediums, including painting, drawing, sculpting, and clay modeling, emphasizing the creative process and the experiential journey.^24^^,^^25^ The primary objective is to amplify the articulation of memories, emotions, and feelings, fostering introspection and facilitating the development and application of novel coping strategies.^24^^,^^31^ While art therapy is extensively applied in treating anxiety among children and adolescents, a unified consensus on its evidence-based outcomes remains absent. Some literature reported positive intervention effects of art therapy for this group of patients,^32^^,^^33^ whereas others contend it lacks distinct advantages over alternative psychological interventions or control groups.^34^^,^^35^ This divergence in findings might be attributed to art therapy's inherent intricacy, versatility, and adaptability, or it could be linked to disparities in aspects like research design, participant count, assessment instruments, treatment duration, or post-treatment observation periods across various studies.^27^

Currently, there is a lack of comprehensive review studies assessing the effectiveness of art therapy in treating anxiety and its specific conditions in children and adolescents. Although we have identified a review focusing on the effectiveness of art therapy in adults, due to the significant differences between adults and children/adolescents in various aspects, these findings cannot be directly generalized. A specific analysis for different age groups is necessary.^36^ When anxiety manifests as the predominant symptom, independent of significant conditions such as cancer or autism, the evidence, and methodologies associated with art therapy's role in alleviating anxiety in this demographic remain ambiguous.^37^ Moreover, the scientific underpinnings that elucidate the anticipated outcomes of the therapy have not been distinctly articulated. Consequently, there is an urgent need to systematically assess the extant literature on the influence of art therapy on anxiety in children and adolescents, scrutinizing its magnitude of effect, contributory factors, and underlying mechanisms.^38^ This study endeavors to conduct a quantitative synthesis of the impact of art therapy on anxiety in children and adolescents through a meta-analysis, aiming to fortify the empirical foundation that buttresses the theory and application of art therapy.

The primary aim of this study is to assess the overall effectiveness of art therapy in reducing anxiety in children and adolescents. The subsidiary aim delves into the distinct modalities of art therapy and contemplates the influence of variables such as patient demographics, duration of the intervention, and study quality on the therapeutic outcomes.

## Methods

This systematic review strictly conformed to the Preferred Reporting Items for Systematic Reviews and Meta-Analysis (PRISMA) guidelines delineated by Moher et al. (2009).^39^ Additionally, this review has been duly registered with the International Prospective Register of Systematic Reviews (PROSPERO), bearing the registration number CRD42023457334.

### Search strategy

A comprehensive literature search was undertaken across various databases on October 22, 2023, to identify relevant studies. The databases included PubMed, Web of Science, Embase (Ovid), PsychINFO, EBSCO, The Cochrane Library (comprising the Cochrane Database of Systematic Reviews and the Cochrane Central Register of Controlled Trials), as well as the Chinese databases including CNKI (China National Knowledge Infrastructure) and Wan Fang Data. To ensure a thorough retrieval, we combined keyword searches with Medical Subject Headings (MeSH) terms: (anxiousness OR nervousness OR hypervigilance OR social anxieties OR anxieties, social anxiety, social OR social anxiety OR angst. MeSH terms: anxiety) AND (painting therapy OR drawing therapy OR sculpture therapy OR poetry therapy OR movement therapy OR dance therapy OR music therapy OR visual art* therapy OR therapies, art OR art therapies OR therapy, art. MeSH terms: art therapy) AND (randomized controlled trial OR randomized OR placebo). Additionally, to bolster the comprehensiveness of this search, we conducted a manual review of the reference lists from the obtained articles, aiming to uncover any further relevant studies.

### Selection criteria

The inclusion criteria for the studies are delineated as follows: First, the study must constitute a Randomized Controlled Trial (RCT). Secondly, participants should span from children to adolescents aged 3‒18, inclusive of all ethnicities and genders. Articles must be published in either English or Chinese. The art therapy modalities can target individual or group sessions without a stipulated duration or session frequency. Eligible studies should incorporate control groups representing inactive treatments (e.g., no treatment, waitlist, sham interventions) or active treatments (e.g., standard care or alternative therapeutic approaches). While co-interventions are acceptable, there must be an assessment of the incremental effect of art therapy on anxiety symptom severity. The primary outcome for consideration in the studies should be the attenuation of anxiety symptoms. Exclusion criteria encompass studies assessing anxiety symptom relief in conditions other than anxiety disorders, those inducing anxiety symptoms in otherwise healthy participants, and interventions that, aside from routine treatment and care, involve other concurrent therapeutic methods. Moreover, studies presenting duplicated, fragmented, or inaccessible data (without consent from the original author) are omitted, as are self-controlled trials, reviews, news articles, conference summaries, editorials, and study guidelines.

### Data extraction

After organizing the search results, a consolidated endnote file was generated, encompassing all the identified references. Duplicate entries were subsequently eliminated. Following this, two independent researchers, ZB and WH, began the study selection process. Initially, they meticulously screened the titles to ascertain their relevance to the research theme. After this preliminary selection, abstracts were rigorously examined, and only those aligning with the inclusion criteria proceeded to a full-text evaluation phase ‒ this thorough appraisal of the complete texts evaluated every article based on predefined eligibility criteria. In cases where discrepancies arose between the two researchers' selections, they sought alignment through discussion or, if necessary, by consulting a third reviewer, AA.

A digital spreadsheet was employed for data extraction, modeled on the Cochrane Collaboration's intervention review data template. This spreadsheet captured pivotal details such as first author, year of publication, demographic characteristics of the sample, numbers in treatment and control groups, detailed description of the art therapy, its duration and frequency, concurrent interventions, control group specifics, measurement instruments, measurement intervals, actual findings of the study. After individual data extraction by the two reviewers, they compared their findings and, following deliberations, reached a unified consensus.

### Quality assessment

Employing the Cochrane Collaboration tool, two reviewers independently evaluated the quality of the included randomized controlled trials for Risk of Bias (RoB) as per Higgins et al. (2011).^40^ The bias assessment encompassed several critical domains: selection bias, performance bias, detection bias, attrition bias, and reporting bias. The risk of bias for each domain was classified into one of three categories: “low”, “high”, or “unclear”. While the RoB tool was initially tailored for pharmacological studies, its application to psychotherapy research necessitated certain modifications, particularly in defining performance bias. In this context, performance bias refers explicitly to “studies that either lacked active control groups or failed to assess patient expectancies and treatment reliability” rather than solely addressing the “blinding of participants and personnel”.^41^

### Statistical analysis

The scores on the anxiety assessment scale are continuous. Although some studies only presented scores for each dimension, they did not provide a comprehensive score for the entire scale. To address this, we extrapolated the overall mean and standard deviation from the Cochrane Handbook for Systematic Reviews of Interventions.^42^ Recognizing the diverse measurement tools employed across the included studies, we assessed using the Standardized Mean Difference (SMD) and its 95% Confidence Interval (95% CI). A p-value below 0.05 was deemed statistically significant for all statistical examinations or model analyses.

In this investigation, given the disparities in interventions, research methodologies, and participant demographics, potential variability exists when estimating genuine effect sizes, a sentiment echoed by Borenstein et al. (2009).^43^ Upon assessing the statistical heterogeneity of the six included RCTs, a Q value of 261.85 emerged, accompanied by a p-value less than 0.01, signifying pronounced heterogeneity amongst these studies. Consequently, the random-effects model was selected for the ensuing meta-analysis. This model acknowledges the heterogeneity between studies, furnishing a more judicious and realistic effect size estimation. In contrast to the fixed-effects model, the random-effects approach offers a superior representation of random errors and true effect size variability across studies, particularly when significant inter-study variation is evident. The synthesized effect size derived serves as an averaged estimation of the actual effects' distribution.

To evaluate the heterogeneity across different studies, we utilized Cochran's Q test.^44^ Furthermore, the I^2^ statistic was invoked to determine the proportion of total variance ascribed to heterogeneity within the studies.^45^ According to Higgins et al. (2003), I^2^ values representing 25%, 50%, and 75% indicate low, moderate, and high heterogeneity levels, respectively. To thoroughly understand and mitigate this heterogeneity, we implemented the random-effects model analysis, sensitivity evaluation, and subgroup analysis, ensuring the robustness and consistency of the present findings.

## Result

### Search results

**Fig. 1:**
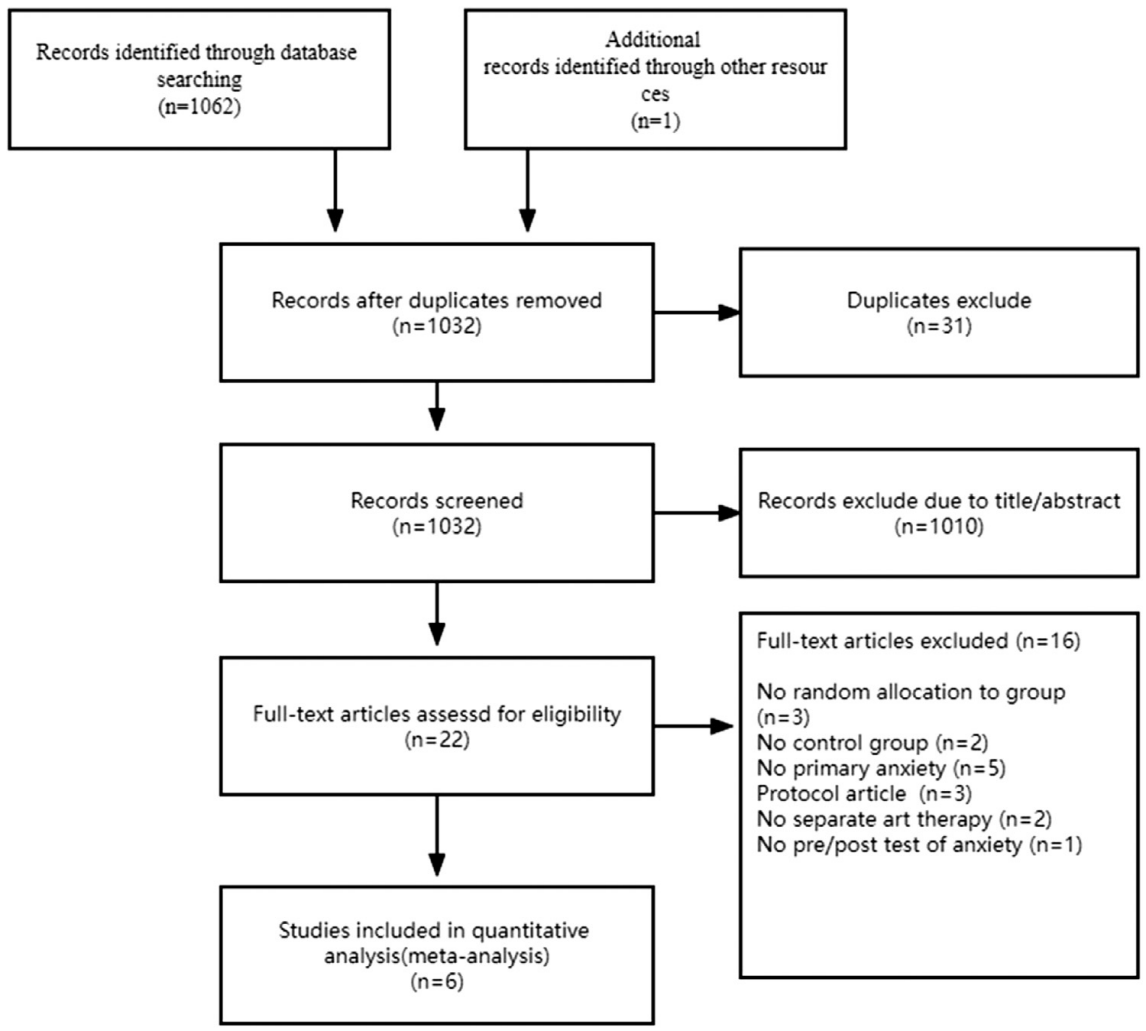
Literature screening flow chart.

From the initially identified 1,063 articles, 31 were discarded due to duplication. The subsequent review of titles and abstracts for the residual 1,010 articles further eliminated 22 articles. A meticulous full-text assessment of these 22 studies led to the dismissal of 16 that needed to align with the inclusion criteria. Factors for exclusion encompassed: lack of randomized controlled trial design, primary outcomes not centered on anxiety symptom reduction, absence of peer-review, deviation from exclusive art therapy, concurrent intervention methods, and inadequate data, among others. In the end, a mere six studies, representing 422 students, satisfied the inclusion benchmarks and were thus integrated into this selection. Fig. 1 offers an expansive breakdown of the screening trajectory.

### Characteristics of included studies

The six articles were published as follows: 2012 (n = 1)^46^ 2012 (n = 1),^47^ 2018 (n = 1),^48^ 2020 (n = 1),^32^ 2022 (n = 2),^33^^,^^49^ as is shown in Table 1. Among the six articles, three are in Chinese and three in English. In terms of geographical distribution, these studies mainly focused on the Asian region, with three studies conducted in China,^46^, ^47^, ^48^ one in India,^32^ one in Iran,^33^ and one in Turkey.^49^ This result may reflect the popularity and acceptance of art therapy in these countries and regions. Of the 422 participants, the experimental group consisted of 213 individuals, while the control group had 209.

**Table 1:** Outcomes and summary of findings from the included studies.

No	Study author & year	Location	Number/ (treated/control)	Study population	Art therapy characteristics	Treatment duration, frequency, type (group or individual)	Co-interventions	Outcome measures	Time points	Intervention(s) and comparator	Significance of outcomes between groups	Significance of outcomes within-groups
1	Günay et al. (2022)^49^	Turkey	62 (32/30)	Sex: Male and female	The activity of making jewelry from beads	Duration: 1.5h	None	STAI-C	Pre- and post-treatment (no follow-up)	Intervention: Making jewelry from beads.	NR	Exp. group: Anxiety (state): S
				Age: 6‒12		Frequency: Twice a week for a total of four weeks						
				Population: Children with cancer						Comparator: Regular activities like drawing, painting, or reading books		Anxiety(trait): S
												Control group: Anxiety(state): NS Anxiety(trait): NS
						Type: Group						
2	Resmy & Raj (2020)^32^	India	30 (15/15)	Sex: Male and female	Dot drawing therapy	Duration: NR	None	Five Facial Anxiety Scale	Pre- and post-treatment (no follow-up)	Intervention: Reading art therapeutic drawing	S	NR
				Age: 7‒12		Frequency: twice a week, in total five weeks						
				Population: Postoperative children								
										Comparator: non-Art restorative		
						Type: Group						
3	Zamanifard et al. (2022)^33^	Iran	40 (20/20)	Sex: Male and female	Virtual-directed painting therapy	Duration: 2h	None	Spence Children's Anxiety Scale	Pre- and post-treatment (no follow-up)	Intervention: Virtual directed painting therapy	S	Exp. group: NS
				Age: 9‒12		Frequency: once a week, in total six weeks						Control group: NS
				Population: Children with type 1 diabetes						Comparator: The routine care provided by the Clinic of Diabetes		
						Type: Group						
4	Huang et al. (2013)^47^	China	60 (30/30)	Sex: Male and female	Sandplay therapy	Duration: 2h	None	CFSS-DS	Pre- and post-treatment (no follow-up)	Intervention: Sandplay therapy	S	Exp. group: S
				Age: 5‒12		Frequency: Once a week, in total four weeks						Control group: NS
				Population: Children with dental phobia						Comparator: The routine care		
						Type: Group						
5	Ma et al. (2018)^48^	China	68 (34/34)	Sex: Male and female	Painting therapy	Duration: 45 min	None	SAS	Pre- and post-treatment (no follow-up)	Intervention: Painting Therapy	S	Exp. group: S
				Age: 15‒18		Frequency: twice a week, in total five weeks				Comparator: General psychological counseling		Control group: S
				Population: Junior high school students with anxiety								
						Type: Group						
6	Wang (2012)^46^	China	70 (35/35)	Sex: Male and female	Music therapy	Duration: 1h	Routine treatment and care	HAMA	Pre- and post-treatment (no follow-up)	Intervention: Music therapy	S	Exp. group: S
				Age: 15‒18		Frequency: twice a week, in total three weeks				Comparator: General psychological counseling		Control group: S
				Population: Junior high school students with anxiety								
						Type: Group						

NR, Not Reported; NS, No Statistical difference; S, Significant; STAI-C, State-Trait Anxiety Inventory for Children; CFSS-DS, Children's Fear Survey Schedule-Dental Subscale; SAS, Zung Self-Rating Anxiety Scale; HAMA, Hamilton Anxiety Scale.

The six RCTs studied diverse populations, ranging from children to adolescents, each grappling with varied anxiety-related challenges. One study pinpointed anxiety in junior high students.^48^ In contrast, the remainder explored children with specific diagnoses, such as cancer, postoperative recovery, Type 1 diabetes, dental phobia, and emotional disorders.^32^^,^^33^^,^^46^^,^^47^^,^^49^ A salient feature across these RCTs is the eclectic array of art therapy modalities harnessed, including painting, sculpture, music, dance, and drama. The studies incorporated various comparator techniques alongside primary art therapy approaches, from casual activities like drawing or reading to structured psychological treatments. Regarding treatment duration and regularity, one study spanned three weeks,^46^ two covered four weeks with sessions twice a week,^47^^,^^49^ two were five-week studies,^32^^,^^48^ and one extended to six weeks.^33^ Of these, four had twice weekly sessions,^32^^,^^49^^,^^46^^,^^48^ while the other two held weekly sessions.^33^^,^^47^ In terms of individual session duration, three studies prescribed 2-hour sessions,^33^^,^^47^ one allocated 1 hour,^46^ another 1.5 hours,^49^ and one settled for 45 minutes.^48^ Another study did not specify timing details.^32^

### Meta-analysis (including overall and sub)

#### Overall analysis

**Fig. 2:**
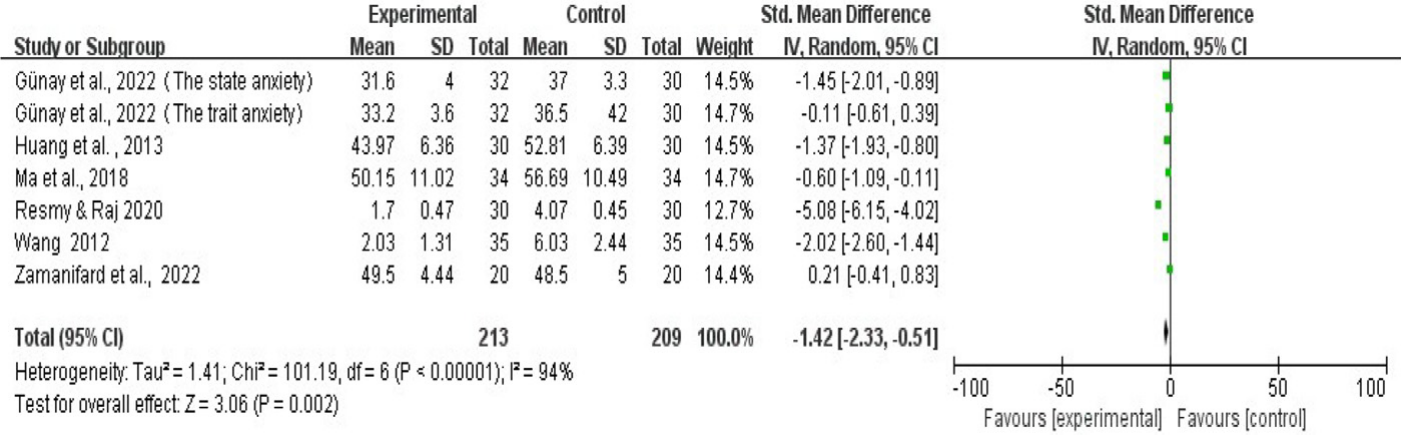
Effects of art therapy on anxiety.

This meta-analysis explored the Standard Mean Difference (SMD) between the experimental and control groups in different sub-studies. It is worth mentioning that the research by Günay et al. (2022)^49^ took into consideration two distinct anxiety dimensions, state anxiety and trait anxiety, and hence, in the present analysis, it was treated as two separate sub-studies. The collective data indicates SMD = -1.42, 95% CI (-2.33, -0.51). This indicates a notable distinction between the experimental and control groups, with the former showing a favorable direction. Further scrutiny demonstrates that the heterogeneity included in the study is significant, confirmed by a Tau^2^ of 1.41, Chi^2^ = 101.19, df = 6, I^2^ = 94%, Z = 3.06, p < 0.002. The overall effect test further substantiates the statistical significance, as is shown in Fig. 2.

#### Detailed sub-group analysis

A deeper examination of the sub-groups revealed two primary research patterns: evaluations once and twice weekly. Weekly Evaluation (Once a week): The data exhibits SMD = -0.59, 95% CI (-2.13, 0.96) for this sub-group. Notably, this sub-group showcases considerable heterogeneity, as indicated by Tau^2^ = 1.15, Chi^2^ = 13.50, and df = 1; moreover, the I^2^ = 93%. Nonetheless, the overall effect for this sub-group is not statistically significant, as the Z = 0.74, p < 0.46.

**Fig. 3:**
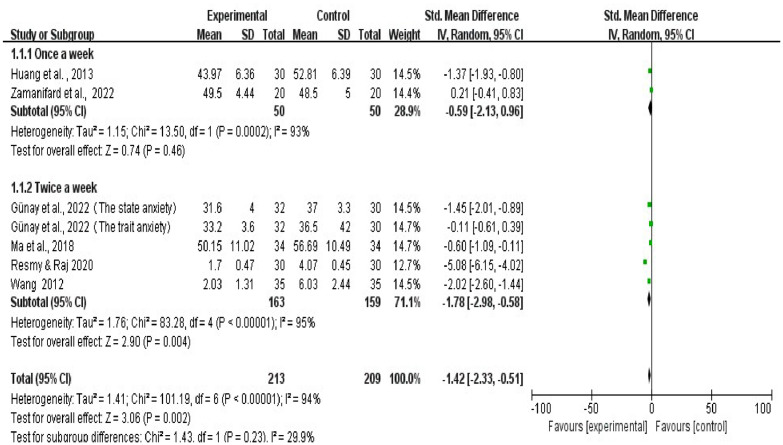
Sub-group analysis of effects of art therapy on anxiety.

Twice weekly Evaluation (Twice a week): In this category, SMD = -1.78, 95% CI (-2.98, -0.58). This group also displays pronounced heterogeneity, evident from a Tau^2^ = 1.76, Chi^2^ = 83.28, df = 4. Additionally, the I^2^ = 95%. Contrary to the once-a-week sub-group, the overall effect for this category is statistically significant, with a Z = 2.90, p < 0.004. These findings are visually represented in Fig. 3.

#### Sensitivity analysis

**Fig. 4:**
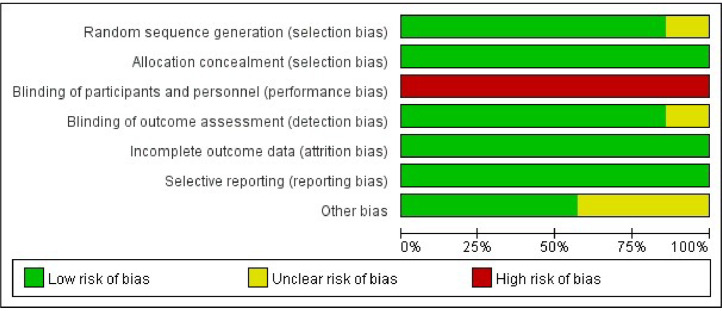
Risk of bias graph.

In contrasting art therapy with control treatments, we executed a sensitivity analysis to meticulously assess the individual impacts of aggregated variables by systematically omitting each study as is shown in Fig. 4. It is pivotal to highlight that, irrespective of the exclusion of particular studies, the direction and magnitude of the amalgamated estimates persisted consistently. This attests to the resilience and robustness of this meta-analysis. Nonetheless, the heterogeneity did not substantially diminish, maintaining a comparatively elevated tier.

### Quality assessment

Using the risk of bias assessment tool provided by the Cochrane Collaboration, we thoroughly evaluated potential biases present in various studies. As presented in the results table, the data conspicuously indicate that most of these studies need to improve in implementing participant blinding. Drawing on the findings of Munder and Barth (2017), the practice of blinding patients and therapists in art therapy mirrors the challenges encountered in psychological treatment studies, which are mainly deemed impracticable. At this juncture, whether the absence of blinding introduced bias into the research outcomes remains ambiguous. Of particular concern is that no studies have assessed treatment expectations or credibility in the initial stages of therapy, leading to these studies being categorized as “high risk” in terms of performance bias. Conversely, the bulk of the other study attributes predominantly aligned with a low risk of bias.

## Discussion

Through a meta-analysis, this study aimed to investigate the impact of art therapy on anxiety among children and adolescents. The findings denote that art therapy considerably ameliorated anxiety symptoms in this demographic. Such an outcome robustly endorses the incorporation of art therapy within the mental health domain, particularly for managing anxiety in children and adolescents individuals. Concurrently, the analysis illuminated that art therapy was more efficacious in alleviating state anxiety compared to trait anxiety. This infers distinct intervention efficacy of art therapy for these two anxiety types. State anxiety typically manifests as ephemeral, context-specific anxiety, whereas trait anxiety aligns more with an individual's enduring predisposition towards anxiety. This insight intimates that art therapy could be particularly effective in helping children and teenagers deal with feelings of anxiety in certain situations.

A deeper exploration of the subgroup data revealed that the study's frequency influenced the efficacy of art therapy. Notably, twice-weekly art therapy sessions manifested a more pronounced effect size than weekly sessions. Such a pattern suggests that amplifying the intervention frequency could potentially bolster the therapeutic impact, particularly in mitigating state anxiety.

The cohort receiving twice-weekly art therapy interventions exhibited a more pronounced decline in anxiety levels compared to the experimental group that engaged in weekly sessions. One plausible rationale posits the sustained impact of art therapy, as the duration of the intervention extends, its potency in diminishing anxiety persists. This aligns with findings from neuroscience research. Kaimal et al. (2017)^50^ observed that after 45 minutes of unstructured artistic activities, participants’ Skin Conductance Response (SCR) markedly reduced and sustained this lower level for an additional 15 minutes. SCR is a physiological metric echoing the autonomic nervous system's activity and emotional arousal intensity. A decline in SCR signifies a state of relaxation and tranquility. Thus, art therapy interventions can be likened to relaxation methods such as meditation or breathwork, facilitating relaxation of both body and mind, subsequently tempering feelings of anxiety in children and adolescents.

This article presents several strengths. Firstly, it employed a systematic search strategy to extract pertinent literature from various databases, applying rigorous inclusion and exclusion criteria to curate appropriate studies. The article also adopted a random-effects model, accommodating the heterogeneity between the studies and yielding a more conservative and authentic effect size estimation. Furthermore, a sensitivity analysis was undertaken, enhancing the reliability and validity of the findings.

While this study offers meaningful insights, several limitations warrant attention. Firstly, given that only six publications satisfied the inclusion criteria, the limited number of studies and small sample sizes compromise the representativeness and extrapolation of the findings. Secondly, the quality of the incorporated studies could have been more consistent, with specific studies presenting methodological shortcomings like the absence of blinding or unspecified randomization techniques, which could introduce biases or errors. Thirdly, the diverse nature, frequencies, durations, and contents of art therapy interventions, coupled with the varied demographics and backgrounds of the participants, might induce heterogeneity, affecting the comparability of results. Lastly, the sole focus on the effects of art therapy on anxiety levels overlooked other pertinent psychological or physiological metrics, such as self-esteem, emotional regulation, life satisfaction, or skin conductance response. These could have provided a more holistic view of the effects of art therapy.

Compared to previous studies, the present findings both corroborate and contradict existing literature. Slayton et al. (2010)^51^ conducted a meta-analysis examining the impact of art therapy on children who have Post-Traumatic Stress Disorder (PTSD) and Co-occurring Anxiety Disorder (CAD). They found a significant impact of art therapy on PTSD but not on CAD. This discrepancy with these findings might stem from the fact that the present study did not exclusively focus on posttraumatic children but included participants with anxiety from various causes. Conversely, Reynolds et al. (2010)^52^ explored the impact of art therapy on the mental health of children and adolescents. Their findings, which highlighted a significant effect on anxiety, align with ours. However, the effect size in the present study was more significant, potentially due to the more frequent and prolonged art therapy interventions the authors employed.

Based on these findings, the authors contend that art therapy has a notable effect in lowering anxiety levels among children and adolescents, bearing critical implications for clinical practice and policymaking. Art therapy is a non-pharmacological, low-risk, and highly acceptable therapeutic method. It offers children and adolescents a safe and creative environment to articulate, comprehend, and regulate their emotions, bolstering their confidence and adaptability. These interventions can also complement other psychological or pharmacological treatments, enhancing the treatment outcomes and prognosis for pediatric anxiety disorders. Moreover, introducing art therapy as a preventative measure in schools, communities, or familial settings can elevate the overall mental well-being of young individuals and mitigate the onset of anxiety.

While the present findings are illuminating, there is still a demand for more rigorous, extensive, and multi-center randomized controlled studies with extended follow-ups to substantiate and broaden these results. Subsequent research should delve into the impacts of varied types, frequencies, durations, and contents of art therapy interventions on pediatric anxiety, taking into account individual variations in age, gender, diagnosis, and background. It is also imperative to understand the underlying mechanisms by which art therapy influences anxiety and other psychological or physiological markers in this demographic. Comparative studies would be valuable, highlighting the strengths and weaknesses of art therapy against other psychological or pharmacological treatments.

## Conclusion

This research highlights the significant role of art therapy in reducing anxiety levels among children and adolescents. These findings have important implications for clinical practices and the formulation of relevant policies. Art therapy, recognized for being non-pharmacological, low-risk, and widely accepted, proves to be an effective method for reducing anxiety symptoms in this population. Nonetheless, the study's limitations must be acknowledged, and the authors anticipate future research to corroborate and enrich these findings. When making therapeutic choices, clinicians and policymakers should weigh various interventions, cater to the unique needs of patients, and aim to enhance the mental well-being of children and adolescents.

## Declaration of competing interest

The authors declare no conflicts of interest.
